# Lumbago

**Published:** 1897-06-12

**Authors:** 


### LUMBAGO.

Slight as are its physical signs, lumbago is a terribly painful disease to the man who suffers from it, and may so completely disable him from certain movements as to render him quite incapable of doing his work. At the same time there is nothing to show for it all—no swelling, no pyrexia, perhaps not even any tenderness, so that it is only by a careful study of the movements of the patient, and by recognising how consistently the pain, the "catch," the sudden check in the movement of the body, repeats itself when the same point is reached, that the physician, when doubt of malingering occurs, can assure himself of the reality of the malady. There are, in fact, as Mr. Hutchinson says, no objective phenomena, unless, indeed, the grimaces and artifices of the patient may be counted as such. This author maintains that although lumbago is commonly described as an affection of the lumbar muscles, that is not the real seat of the pain, nor, indeed, is it in the loins at all. If we wish to define it with precision, we should, he says, understand the word to denote a liability to pain on movement felt across the sacrum, or in the sacro-iliac synchondroses, as a rule on both sides.

Free from all danger as lumbago may be, it is a malady which no patient can afford to make light of, not merely from the severity of the pain, but on account of the suddenness with which its attacks are apt to come on, and the complete incapacity for work which they often involve until they pass away.

Once experienced, the liability of recurrence of the attacks remains, affected no doubt, like other gouty things, by weather, diet, and various causes, but still continuing often for the rest of life.

Sometimes the liability to attack only occurs at certain seasons, but whenever the patient is conscious of the tendency, he will have to move carefully, will have to avoid strange attitudes, and, above all, will never have to remain in any one attitude for long, however comfortable it may seem, or he will risk a paroxysm.

In the milder cases the curative measures are active exercise, care in diet, and warm clothing, especially across the hips. Mr. Hutchinson particularly insists that in such cases the patient should, if possible, move about even during the pain. It may be that at first he can only get about with assistance, and bent almost double; but he should persevere in moving his back in all directions which are possible to him, and after a time he will find that the pain will lessen, and the range of movement will become greater.

In more severe cases counter-irritants and the local application of heat will be found useful, while aconite, alkalies, and colchicum may be given internally.

In both lumbago and sciatica, in fact in all forms of rheumatic gout, the free employment of hot weak tea is advised as one of the most effectual remedies.

